# Measuring the neutral zone of spinal motion segments: Comparison of multiple analysis methods to quantify spinal instability

**DOI:** 10.1002/jsp2.1088

**Published:** 2020-04-25

**Authors:** Theodor Di Pauli von Treuheim, Olivia M. Torre, Grace E. Mosley, Philip Nasser, James C. Iatridis

**Affiliations:** ^1^ Leni & Peter W. May Department of Orthopaedics Icahn School of Medicine at Mount Sinai New York New York USA

**Keywords:** intervertebral disc, load‐deflection curve, neutral zone, spinal instability, spine biomechanics

## Abstract

**Purpose:**

Neutral zone (NZ) parameters in spinal biomechanics studies are sensitive to spinal instability, disc degeneration, and repair. Multiple methods in the literature quantify NZ, yet no consensus exists on applicability and comparability of methods. This study compares five different NZ quantification methods using two different load‐deflection profiles.

**Methods:**

Rat caudal and lumbar motion segments were tested in axial rotation to generate load‐deflection curves with profiles exhibiting prominent distinction between elastic and NZ regions (ie, triphasic) and profiles that did not (ie, viscoelastic). NZ was quantified using five methods: trilinear, double sigmoid (DS), zero load, stiffness threshold (ST), and extrapolated elastic zone. Absolute agreement and consistency of NZ parameters were assessed using intraclass correlation (ICC), Bland‐Altman analyses, and analysis of variance.

**Results:**

For triphasic profiles, NZ magnitude exhibited high consistency (methods correlate but differ in absolute values), and only some methods exhibited agreement. For viscoelastic profiles, NZ magnitude showed limited consistency and no absolute agreement. NZ stiffness had high agreement and consistency across most methods and profiles. For triphasic profiles, the linear NZ regions for all methods were not well‐described by a linear fit yet for viscoelastic profiles all methods characterized a linear NZ region.

**Conclusion:**

This NZ comparison study showed surprisingly limited agreement and consistency among NZ parameters with approximately 5% to 100% difference depending on the method and load‐deflection profile. Nevertheless, the DS and ST methods appeared to be most comparable. We conclude that most NZ quantification methods cannot be applied interchangeably, highlighting a need to clearly state NZ calculation methods. Future studies are required to identify which methods are most sensitive to disc degeneration and repair in order to identify a “best” method.

## Graphical abstract

The neutral zone (NZ) is a clinically relevant metric of spinal motion segment laxity, but measuring it is difficult due to the viscoelastic nature of the intervertebral disc. Various NZ quantification methods are currently used in the field, yet the degree of agreeability and interchangeability between methods remains unknown. Our findings indicate that methods rarely agree in measurement and should therefore not be considered interchangeable, highlighting the need to clearly describe the underlying methodology in order to contextualize NZ results.

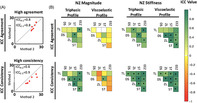

**Abbreviations**
DS: double sigmoid method
EEZ: extrapolated elastic zone method
NZ: neutral zone
ST: stiffness threshold method
TL: trilinear method
ZL: zero load method

## 1 INTRODUCTION

Lumbar spine instability is widely regarded to be an important factor involved in chronic back pain.^1^, ^2^, ^3^ Measuring spinal instability experimentally informs clinical decision‐making and improves understanding of degenerative changes and injury mechanisms.^4^, ^5^, ^6^ The load‐deflection behaviors of spinal motion segments are highly nonlinear with minimal stiffness around the neutral position and increasing stiffness as motions increases; this nonlinearity provides resistance and prevents damage toward the limits of the load‐deflection curve.^1^, ^7^, ^8^, ^9^ Panjabi defined the neutral zone (NZ) as the portion of the spinal motion load‐deflection curve where motion is produced with a minimal resistance. NZ and range of motion are commonly calculated parameters in biomechanical studies employed to characterize the two major portions of this nonlinear load‐deflection curve and assess motion segment instability. The NZ can be more sensitive than range of motion in characterizing instability and is a clinically relevant biomechanical metric.^1^, ^3^, ^7^, ^10^, ^11^ NZ is also commonly measured during biomechanical assessment following disc repair strategies and when characterizing material properties.^12^, ^13^

**FIGURE 1:**
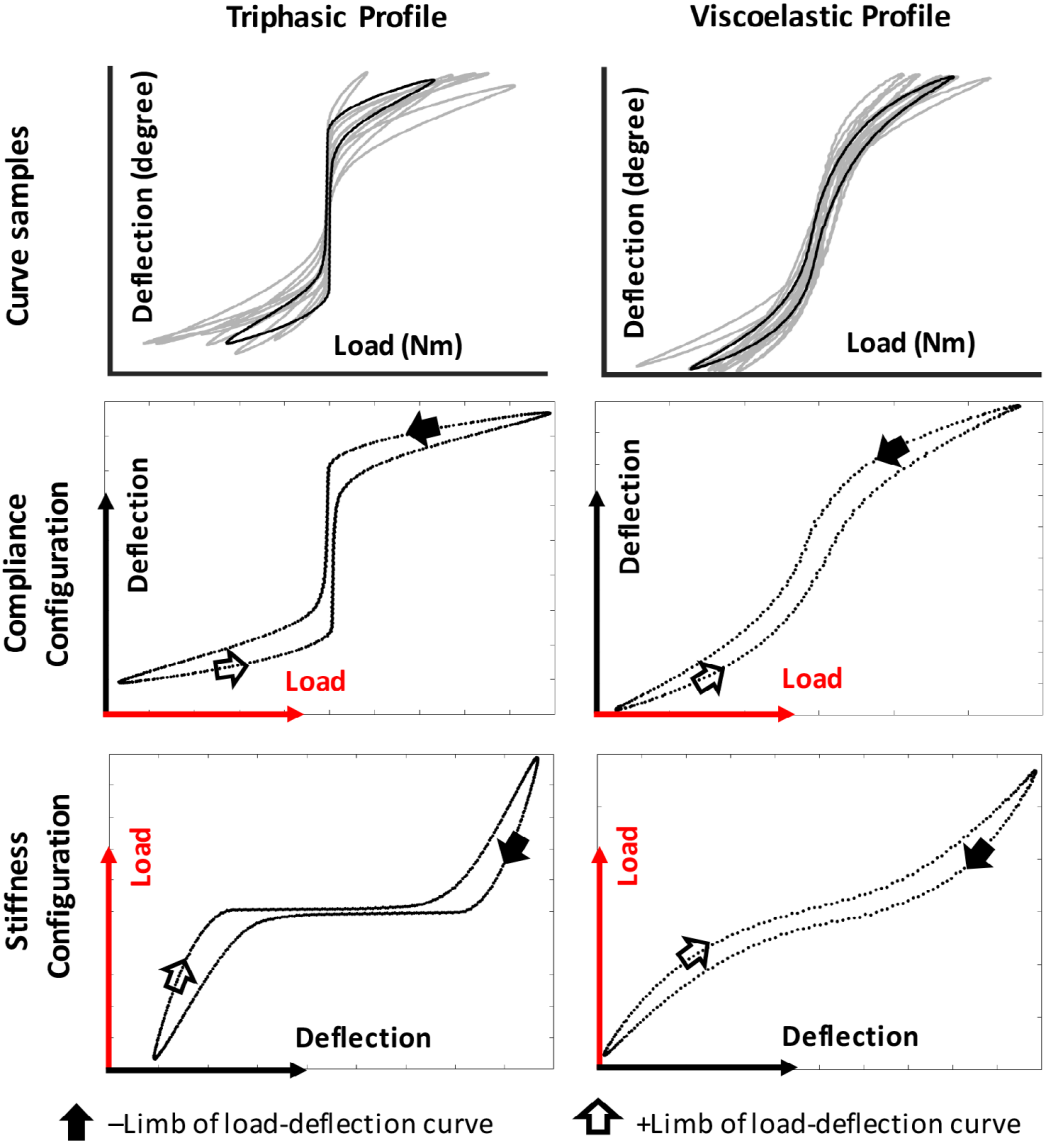
Stiffness and compliance configurations for triphasic and viscoelastic load‐deflection profiles. Biomechanical testing was performed using a stiffness protocol, and data was transformed to flexibility configuration whenever neutral zone (NZ) quantification methods required this configuration (ie, double sigmoid and zero load NZ quantification methods). The trilinear, stiffness threshold, and extrapolated elastic zone NZ quantification methods required data in the stiffness configuration. Triphasic load‐deflection profiles exhibit a prominent transition from NZ to elastic regions while viscoelastic load‐deflection profiles have more subtle transition from NZ to elastic region and exhibit a more prominent viscoelastic hysteresis. Arrows indicate the progression of motion through one testing cycle

While the conceptual definition of NZ is well accepted, consensus on the choice of mathematical definition for calculating NZ parameters is lacking. To date, no study has investigated the differences of NZ quantification methods to determine whether they are similar or interchangeable. Current methods employed to measure the NZ from load‐deflection curves include the trilinear (TL),^14^, ^15^ double sigmoid (DS),^8^ zero load (ZL),^7^ stiffness threshold (ST),^16^ and extrapolated elastic zone (EEZ)^11^ methods. The names of these methods either exist in the literature or were defined for the purposes of comparison in this study. Each method employs distinct calculations to define the location of NZ boundaries that constrains the NZ region for a given load‐deflection curve. The load‐deflection curve is comprised of loading in the positive direction (+limb) and negative direction (−limb). Methods define the boundaries constraining the NZ region of the load‐deflection curve in order to calculate NZ magnitude and NZ stiffness. Some NZ calculation methods were developed using a stiffness configuration (ie, y‐axis describes deflection and the x‐axis describes load), while others necessitate a compliance configuration (ie, y‐axis describes load and the x‐axis describes deflection) (Figure 1). As presented in their original papers, the TL, ST, and EEZ methods require the stiffness configuration, whereas DS and ZL methods require the compliance configuration. Further complicating NZ calculations are the variations in load‐deflection curves that vary in their profile depending on testing conditions and motion segment properties. Some load‐deflection profiles exhibit a prominent elastic and NZ region (termed triphasic), while others have a more subtle transition from NZ to linear elastic region, yielding load‐deflection curves which exhibit a more gradual viscoelastic hysteresis characteristic (termed viscoelastic) (Figure 1).

**FIGURE 2:**
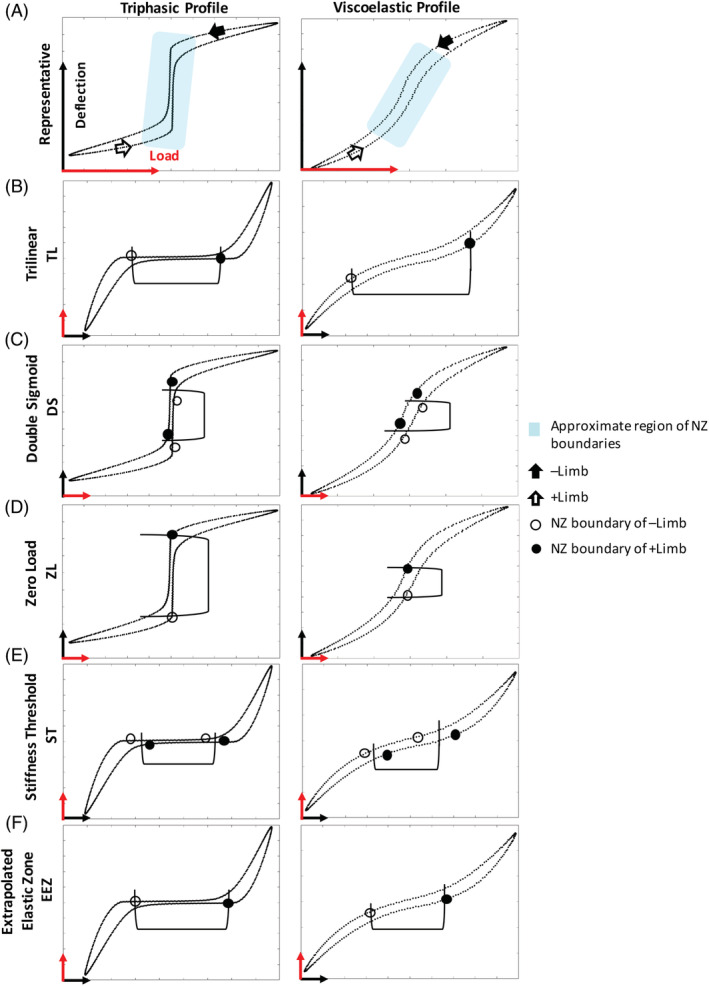
Methods generate neutral zone (NZ) boundaries, which are used to calculate NZ magnitude and NZ stiffness. A, Representative load‐deflection curves of triphasic and viscoelastic profiles are separated by columns and the region in which the NZ was expected to lie was highlighted in blue. Stiffness vs flexibility configurations are indicated by black/red arrows. NZ boundaries are demarcated with filled and unfilled dots. Trilinear (TL), zero load (ZL), and extrapolated elastic zone (EEZ) methods generate one NZ boundary for each limb. Double sigmoid (DS) and stiffness threshold (ST) generate bilateral NZ boundaries for each limb (two filled and unfilled dots for both limbs). The final NZ boundary (bracket) is the average of NZ boundaries at either pole. The difference in deflection represents the NZ magnitude. Representative calculations are shown for B, TL; C, DS; D, ZL; E, ST; and F, EEZ methods

The TL fitting method was first described by Sarver et al. for analysis of axial tension‐compression loading curves in a mouse model.^14^ This method was subsequently amended to load‐deflection curves with less abrupt transition breakpoints.^15^ In the stiffness configuration, a third‐ or fifth‐degree polynomial is fitted to the loading portion of the +limb and ‐limb, neglecting the unloading portion of the curve.^15^, ^17^ The point of minimum slope of the fitted polynomial is derived, and a tangent line extrapolated. Next, a line is fitted to the elastic zone (80%‐100% of maximum load) of both loading limbs. The intersection of the elastic zone line with the tangent line generates coordinates through which lines are extended parallel with the y‐axis. Intersections of these parallel lines with both limbs of the load‐deflection curve yield the NZ boundaries (Figure 2B).

The DS method was described by Smit et al as a purely algorithmic method without arbitrary stipulations and assumptions.^8^ The DS function is composed of 10 parameters that are iteratively adjusted and fitted to the experimental data using unconstrained nonlinear minimization of the root mean square error. Both limbs are fitted with the DS function in the compliance configuration, and first and second derivatives are computed from the fitted curve. Physiologically, the second derivative explains the change in intervertebral disc compliance along the fitted load‐deflection curve. Compliance, being the inverse of stiffness, is highest within the NZ region and the inflection points produced by the second derivative provide mathematical endpoints of this region (Figure 2C). These endpoints are determined for both limbs, and their averaged coordinates create the NZ boundaries.

The ZL method was published by Wilke et al and it requires that axes be in the compliance configuration.^7^ The NZ is defined as the difference in deflection between both directions of the load‐deflection curve at zero load. This method does not generate NZ boundaries along the load‐deflection curve. Instead, the NZ magnitude projects orthogonally between curve limbs (Figure 2D). NZ boundaries are generated by extending a line through these points parallel to the x‐axis (load axis) until intersecting with both limbs of the load‐deflection curve.

The ST method was originally described by Thompson et al, where the ST was explicitly predefined at ±0.05 N/° with the load‐deflection curve in the stiffness configuration.^16^ This method was amended in our study so that the ST would depend on a baseline load‐deflection curve stiffness value. The baseline value was obtained using a computational algorithm that scanned a linear segment along a limb of the load‐deflection curve searching for the lowest stiffness fit. The slope of the segment represents the baseline and is consequently extrapolated bidirectionally along the limb of the load‐deflection curve until the baseline stiffness deviates by no more than 5%. The endpoints of the extrapolated segment generate the NZ boundary. NZ boundaries for both directions are then averaged (Figure 2E).

Both Gay et al and Spenciner et al published studies using the EEZ method.^11^, ^18^ In the stiffness configuration, a line is fit to the elastic zone (80%‐100% of maximum load) of both limbs, similarly to the TL method. However, the elastic zone lines are extrapolated until intersecting with the x‐axis. The NZ boundaries are found by extending a line, in parallel with the y‐axis, from these intersections through the load‐deflection curve (Figure 2F).

NZ quantification methods are commonly used interchangeably across the literature. These five methods employ different strategies and assumptions when generating NZ boundaries and could therefore yield dissimilar NZ values depending on the shape of the load‐deflection curve. The current study included “triphasic” load‐deflection curve profiles that have abrupt breakpoints, and “viscoelastic” load‐deflection curves that have more subtle breakpoints in the transition from NZ to elastic region. Specimens were tested in axial rotation due to its known sensitivity in measuring NZ instability.^10^, ^13^, ^19^, ^20^ The main objective of this study was to compare NZ parameters generated by the NZ quantification methods (TL, DS, ZL, ST, and EEZ) using analysis of variance (ANOVA), intraclass correlation, and Bland‐Altman analyses to determine agreement, consistency and percent differences between methods. A secondary objective was to determine if the NZ boundaries constrained a linear NZ region, thus permitting calculation of a constant stiffness NZ. This study did not calculate the range of motion, elastic stiffness, or other biomechanical parameters, which are known to have greater measurement consistency, and instead focused on NZ parameters, which traditionally yield the largest variance. It was hypothesized that NZ quantification methods may occasionally be dissimilar, but that differences in measurements would be proportional and independent of profile shape.

## 2 MATERIALS AND METHODS

### 2.1 Specimen preparation

Lumbar (L5/6) and caudal (c4/5, c6/7, c8/9) motion segments from skeletally mature Sprague Dawley rats were dissected with intact rostral and caudal vertebrae and stripped from spinal ligaments and posterior segments. Motion segments were then covered in phosphate‐buffered saline (PBS)‐wetted Kim wipes and stored at −80°C (lumbar) and −80°C (caudal). On testing day, specimens were thawed in 1X PBS for 2 hours and potted in stainless steel cylindrical pots with Loctite 401 cyanoacrylate (Henkel, Düsseldorf, Germany), as previously described.^21^, ^22^ Intact rat caudal motion segments (n = 7) were selected because they reliably display triphasic load‐deflection curve profiles, while intact rat lumbar motion segments (n = 10) were selected for their viscoelastic load‐deflection curve profiles.^23^ All animal procedures were approved by the Institutional Animal Care and Use Committee and Icahn School of Medicine at Mount Sinai.

### 2.2 Testing conditions

Motion segments were secured into a torsional testing machine (AR2000ex, TA Instruments), preconditioned in axial compression at 0.5 N for 5 minutes, and subjected to 20 cycles in axial rotation following previously established protocols.^21^, ^22^ For caudal motion segments, rotation of ±20° at a frequency of 0.5 Hz was chosen to obtain load‐deflection curves that captured linear regions in the clockwise and counterclockwise directions. Lumbar motion segments were rotated to a maximum of ±10° at a frequency of 1 Hz.^22^ The load‐deflection curve of the 20th cycle was used for analysis.

### 2.3 Replication of NZ quantification methods

All NZ methods were replicated following instructions and descriptions from the literature and aggregated into a custom‐made MATLAB Graphical User Interface program, which computed the desired outputs for any input dataset (Release 2018b Mathworks Natick, Massachusetts). For the TL method, only the third‐degree polynomial was used because the fifth‐degree polynomial did not enhance the fit for our dataset's load‐deflection curves. Both polynomial fits were previously used interchangeably depending on the fit to the load‐deflection curve.^15^, ^17^ The polynomial was fit to the data using the MATLAB curve fitting toolbox function, optimizing by linear least squares. For the DS method, optimization of the 10 parameters was achieved by applying a profile‐dependent template DS curve to a given load‐deflection data. The starting conditions could then be optimized prior to curve fitting, which reduced the number of function evaluations required for the curve fitting optimization procedure. MATLAB curve fitting toolbox functions were used, minimizing by nonlinear least squares. In our experience, this methodology was easier to apply, more robust, and produced the same results as the “fminsearch” MATLAB function, which was used by Smit et al.^8^ Next, the 10 parameters from the optimization procedure were incorporated into a symbolic representation of the DS function, permitting calculation of first and second derivatives to determine inflection points. ZL, ST, and EEZ methods did not require optimized curve fitting (Figure S1).

TL, ZL, and EEZ methods generate a single NZ boundary per limb. In order to compare uniformly between methods, NZ boundaries were obtained for both limbs of the load‐deflection curve. This was accomplished by extending lines in parallel with the load axis from the single NZ boundary until both limbs of the load‐deflection curve were intersected. Contrarily, DS and ST generate one set of NZ boundaries per limb. These boundaries were averaged on both poles and a line was extended in parallel with the load axis to generate the final NZ boundaries (Figure 1B). This procedure has previously been reported.^8^

NZ magnitude and NZ stiffness were calculated from NZ boundaries obtained from each replicated method. The angular displacement between the NZ boundaries represents the NZ magnitude. Definitions of NZ stiffness differ in the literature and often depend on the aims of a particular study. In this study, NZ stiffness refers to the slope calculated from the secant line connecting NZ boundaries, which permitted standardization for comparisons within the study. NZ stiffness was calculated for both limbs with the load‐deflection curve in the stiffness configuration.

### 2.4 Statistical and NZ comparison analyses

Statistical analyses were performed using MATLAB and SPSS (SPSS Statistics 24, IBM, USA) where *P* < .05 was significant. All data were normally distributed as determined by a Shapiro‐Wilk Normality test. The NZ boundaries were interpreted for comparisons of pooled NZ parameters, comparisons, and agreement, as well as assessment of NZ region linearity. Boxplots were constructed to demonstrate the distribution of the pooled values with median, IQR between the 25th and 75th percentiles, and whiskers extending to the most extreme values not including outliers. One‐way ANOVA with post hoc Holm‐Bonferroni corrected multiple comparison tests determined the effects between NZ methods. Absolute agreement (ICC_A‐1_) and consistency (ICC_C‐1_) of NZ magnitude and NZ stiffness were assessed between methods using a two‐way, mixed, single measures intraclass correlation analysis. Absolute agreement accounts for fixed and proportional differences, whereas consistency reflects only proportional differences between methods (Figure 4A).^24^, ^25^ These tests are more appropriate than Pearson's coefficient of correlation when comparing between methods that are designed to measure the same entity.^26^ ICC values were displayed with heat maps. Lastly, NZ magnitude and NZ stiffness limits of agreement were visually contextualized using Bland‐Altman analyses.^27^, ^28^ Bland‐Altman plots are commonly used to assess interchangeability between methods, where the difference between methods is plotted against their average. Limits of agreement denote ±2SD of the dataset. If limits of agreement comparing two methods remained within the maximum acceptable difference of 30%, then the comparison was interpreted to be interchangeable (Figure 5A). Lastly, a sequential polynomial regression analysis with linear and cubic models was calculated on the region constrained by the NZ boundaries in order to inform the relationship between load and deflection generated by a method. Assessing the NZ for its linearity informed whether NZ stiffness, measured as the slope of the linear fit of this region, could be assumed an accurate approximation of the load‐deflection behavior in this NZ region. It was expected that wide NZ boundaries would lead to a poor fit with a linear model and be enhanced with a cubic model (Figure 6A). Linear and cubic models were quantified by their R^2^ values.

## 3 RESULTS

### 3.1 Neutral zone magnitude differed between all methods within viscoelastic profiles but was more comparable within triphasic profiles

**FIGURE 3:**
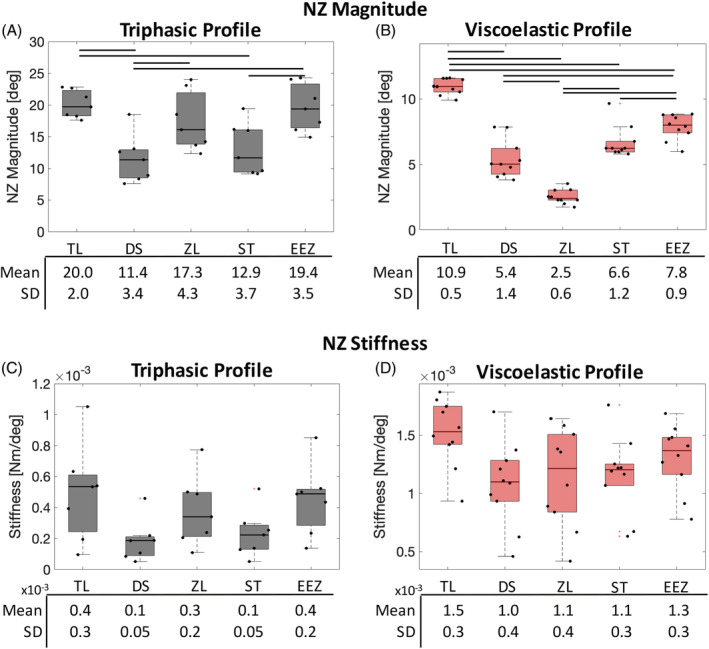
Neutral zone (NZ) magnitude and NZ stiffness are compared across NZ quantification methods for triphasic and viscoelastic loading profiles. Boxplots represent the median and 25%ile to 75%ile of data. Mean and SD shown below. One‐way analysis of variance with Holm‐Bonferroni corrected post hoc comparisons are marked with a line if statistically significant (*P* < .05). A, Significant differences exist with NZ magnitude for both profiles with greater differences determined in the viscoelastic cohort. B, No differences were observed between methods for NZ stiffness

The NZ magnitude values differed significantly between methods in both profile cohorts (Figure 3A). Within the triphasic cohort, comparisons of NZ magnitudes from TL, ZL, and EEZ methods were not significantly different, and neither were values between DS and ST methods. However, in the viscoelastic cohort, NZ magnitude calculated from all method comparisons differed significantly. The within method variability for NZ magnitude was larger for the triphasic profile cohort than the viscoelastic cohort. The relationship between methods of pooled values was similar across profile type except for the ZL method, which had the smallest NZ magnitude for viscoelastic profiles vs third largest NZ magnitude for triphasic profiles. Post hoc analyses showed that NZ magnitude calculated with the TL method most frequently differed to the other methods across both profile types, where it was significantly different to all other methods when applied to viscoelastic profiles. The choice of method had large effects on the NZ values. Mean NZ values for the triphasic cohort ranged from 11.4° to 20.0°, corresponding to 28% to 50% of the applied ROM (±20°). On the other hand, mean NZ values for the viscoelastic cohort ranged from 2.5° to 10.9°, corresponding to 12.5% to 54% of the applied ROM (±10°).

There were no differences in NZ stiffness between methods for either profile cohort (Figure 3B).

### 3.2 Neutral zone magnitude and neutral zone stiffness measurements differed in consistency and agreement between triphasic and viscoelastic profiles

**FIGURE 4:**
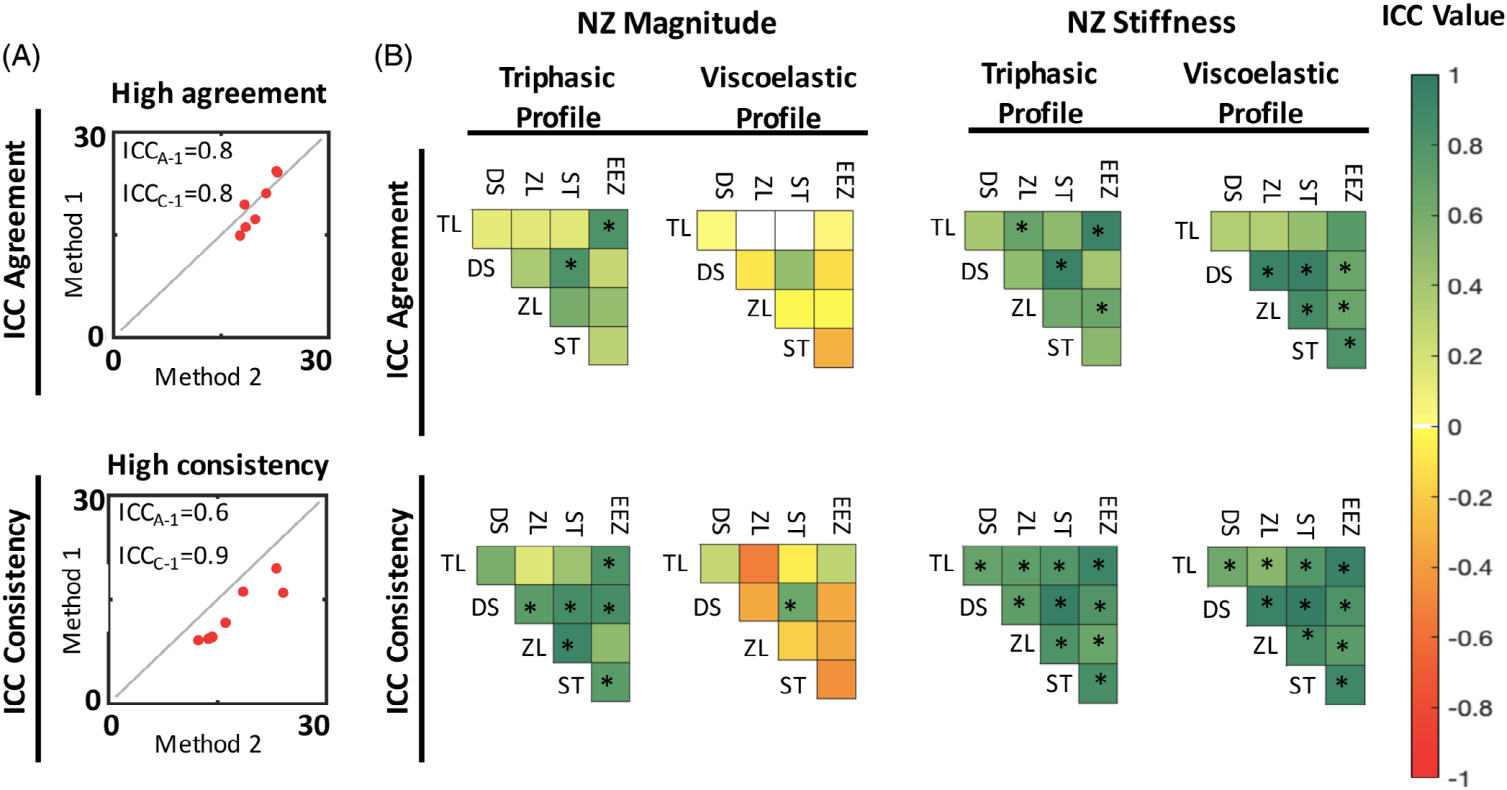
Agreement and consistency of neutral zone (NZ) magnitude and NZ stiffness calculations using univariate intraclass correlation analyses. A, Representative comparison showing absolute agreement (capture of both fixed and proportional differences) and consistency (capture of only proportional differences). B, Agreement and consistency heatmap matrices of NZ magnitude (left) and NZ stiffness (right) correlate all methods for both profile cohorts. Intraclass correlation (ICC) values of +1 indicate perfect correlation, while negative values show an inverse relationship between methods. Statistically significant (*P* < .05) ICC correlation values are marked by an asterisk indicating that the correlation is not due to chance

**FIGURE 5:**
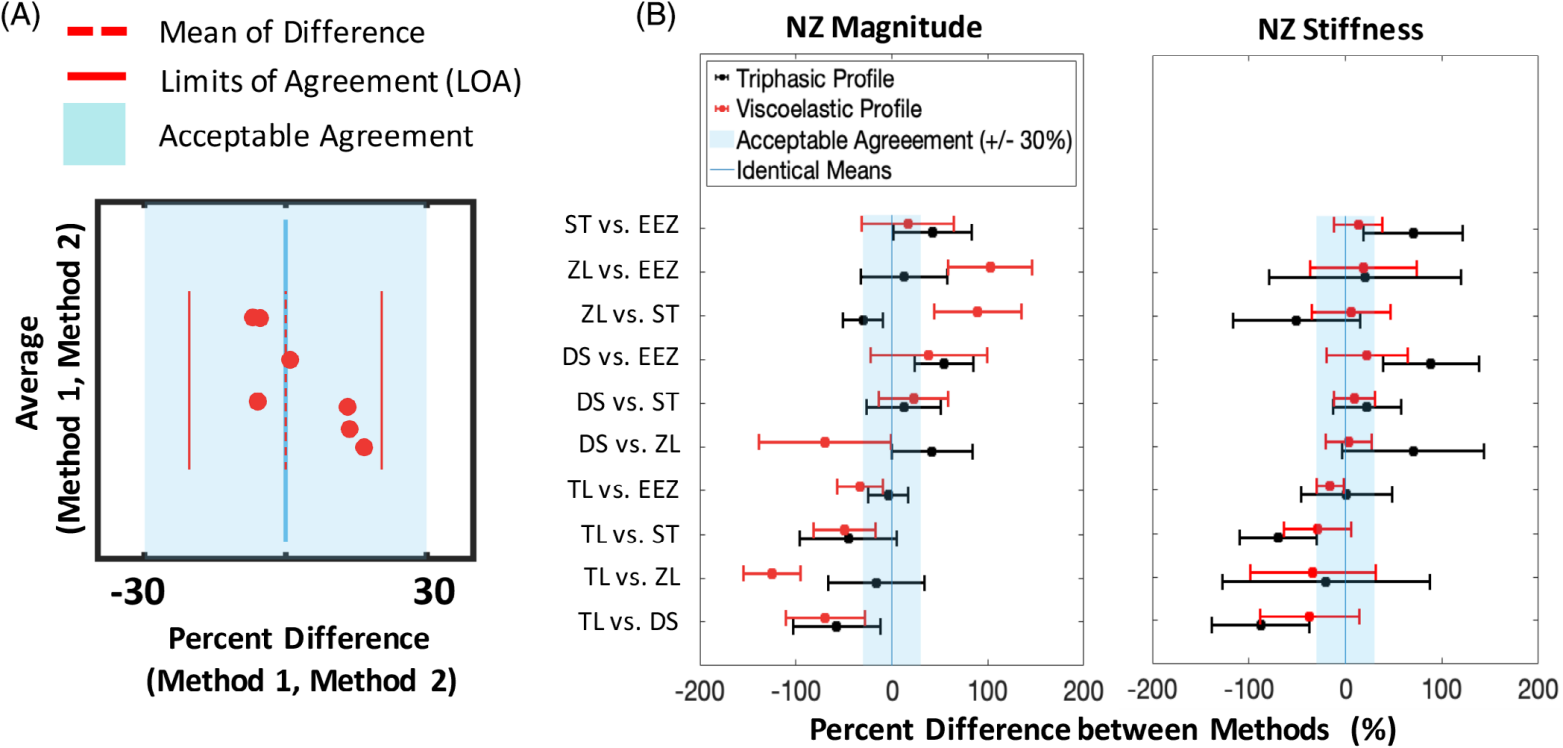
Relative agreement between neutral zone (NZ) parameters from different methods using Bland–Altman plots. A, A representative Bland‐Altman plot shows the difference between methods on the x‐axis plotted against their average, since no gold standard method was assumed (the y‐axis). A liberal threshold was used for the maximum acceptable difference region, set within 30%. By convention, limits of agreement are calculated as ±2 SD from the mean. B, Condensed Bland‐Altman plots illustrate all comparisons for NZ magnitude and NZ stiffness with y‐axis values from panel A averaged and indicated by a dot

In general, NZ magnitude agreement results from the triphasic profile cohort did not parallel results from the viscoelastic cohort. Across both profile cohorts, only DS‐ST methods agreed in the triphasic cohort while also correlating consistently in the viscoelastic cohort with an ICC consistency of 0.7 (*P* = .014) (Figure 4B). NZ magnitude agreement and consistency between methods was more frequent in the triphasic cohort. In this cohort, significant NZ magnitude ICC agreement was observed between TL‐EEZ and DS‐ST (Figure 4 **B**). Bland‐Altman analysis showed limits of agreement, which remained within 30% maximum acceptable difference only for TL‐EEZ, while DS‐ST produced limits of agreement that differed up to 51% (Figure 5B). Comparisons of methods DS, ZL, and ST produced significant NZ magnitude ICC consistency correlations in the triphasic cohort. DS and ST also achieved consistency with the EEZ method, demonstrating relatability between more methods. The TL method was only significantly consistent with the EEZ method (Figure 4B). Agreement and consistency were rare in the viscoelastic cohort, with some methods correlating negatively. Bland‐Altman results show wide limits of agreement that never remained within the 30% maximum allowed difference region and frequently crossed beyond 100% difference indicating these methods are not interchangeable (Figure 5B).

Higher absolute agreement and consistency were observed for NZ stiffness compared to NZ magnitude for both curve profiles (Figure 4). NZ stiffness ICC values were significantly consistent for all method comparisons in both profile cohorts; however, significant NZ stiffness agreement was only achieved in both cohorts between DS‐ST and ZL‐EEZ. In the viscoelastic cohort, all method comparisons excluding TL generated significantly agreeing ICC values. In this cohort, the Bland‐Altman NZ stiffness plot visually conveyed that only DS‐ST and DS‐ZL generated limits of agreement within the 30% maximum acceptable difference region (Figure 5B). The rationale behind these large limits of agreement is that the NZ stiffness has very small magnitudes. Additionally, the relationship between TL‐EEZ became apparent as one with tight limits of agreement and fixed bias, implying that interchangeability between these methods may not be appropriate due to the consistent overestimation of one method over the other. The NZ stiffness Bland‐Altman plots for the triphasic profile cohort illustrated large limits of agreement that did not remain within 30% of the maximum acceptable difference and in some cases approached more than 100% difference (Figure 5B).

### 3.3 All methods determined a similar linear neutral zone region

**FIGURE 6:**
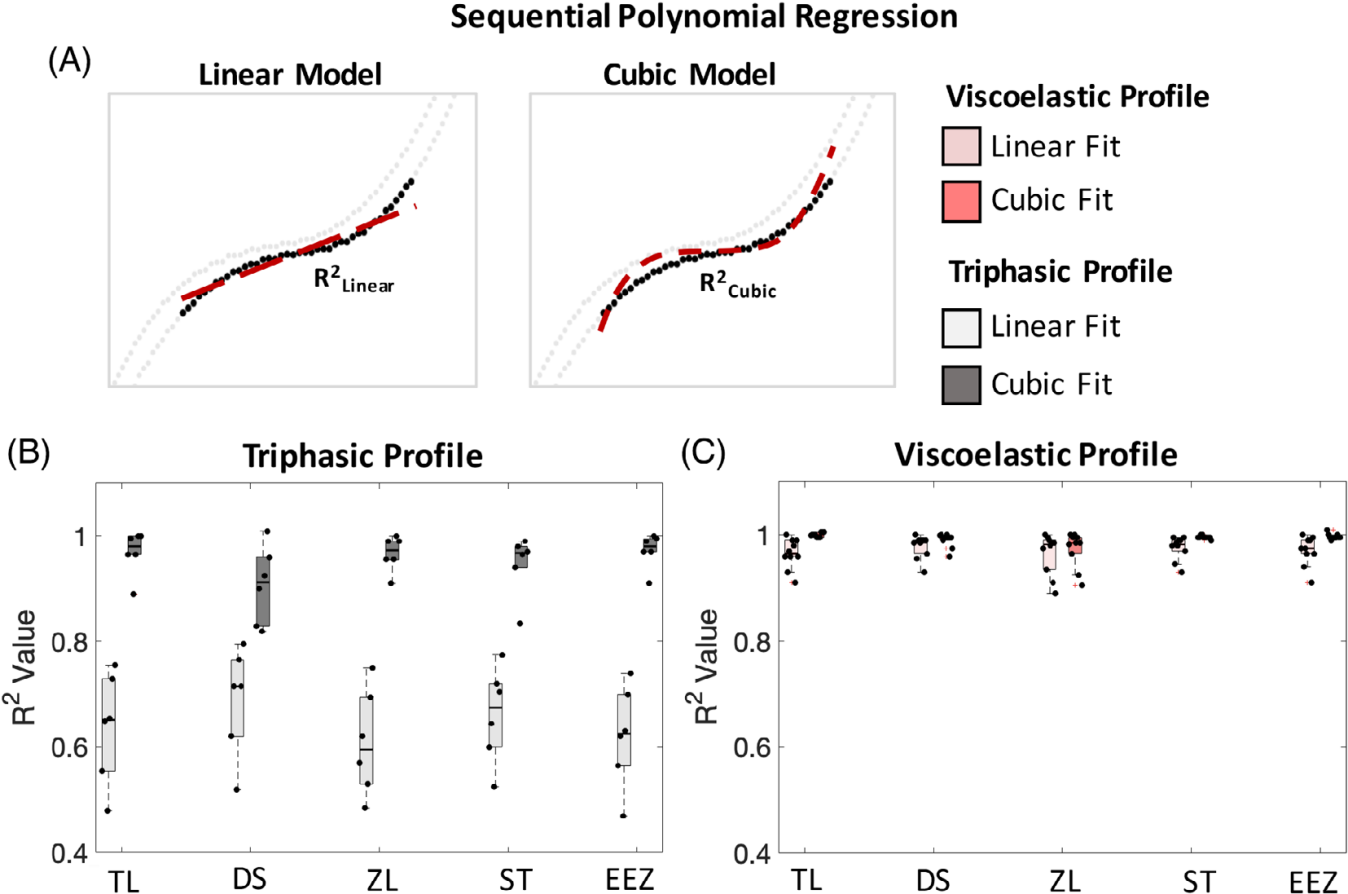
Analysis of the relationship between load and deflection within the neutral zone (NZ) region. Sequential polynomial regression analysis quantified the NZ region using linear and cubic models. A, The NZ region (dark black data points) was characterized to assess the linearity of the isolated NZ region (gray data points indicate elastic region). Coefficients of determination R^2^ were pooled and compared between NZ quantification methods using boxplots. Each limb was analyzed separately. B, The triphasic cohort had R^2^ values that were substantially greater for the cubic than linear models, highlighting the nonlinear NZ region slope. C, The viscoelastic cohort had R^2^ values well above 0.9 for all NZ quantification methods indicating a linear NZ region

All methods were able to generate NZ boundaries within the visually expected region. Methods constrained boundaries that produced higher linear R^2^ values for viscoelastic compared to triphasic profiles (Figure 6B). In the triphasic profile cohort, all methods generated NZ boundaries for which a linear fit model explained on average 60% to 68% of the variance in the NZ region (mean R^2^_linear_: TL = 0.63; DS = 0.68; ZL = 0.60; ST = 0.66; EEZ = 0.62). When a cubic model was used to fit the NZ data, it explained an additional 22% to 36% of the variance (mean R^2^_cubic_: TL = 0.96; DS = 0.90; ZL = 0.96; ST = 0.94; EEZ = 0.97). The greatly improved fit of cubic function to the NZ load‐deflection data indicates that the triphasic NZ region is best described by a non‐linear function, and that the linearity assumption for the NZ stiffness calculation may therefore not be valid. For viscoelastic profiles, the linear assumption was adequate. All methods generated NZ boundaries for which the linear model explained greater than 96% of the variance (median R_linear_^2^: TL = 0.97; DS = 0.98; ZL = 0.96; ST = 0.98; EEZ = 0.97) and the cubic model did not substantially increase the R^2^ value. In both profile cohorts, the DS method generated NZ boundaries with the highest linear model R^2^ value, suggesting that it best defined a linear NZ region. Nevertheless, this result was not statistically different in comparison to the other methods.

## 4 DISCUSSION

Quantifying changes in spinal motion segment stability remains a critical priority in spinal biomechanics, yet the highly nonlinear nature of load‐deflection profiles makes precise quantification of the NZ challenging. The equivalency of current NZ quantification methods remains unclear. This study compares NZ parameters obtained from the five most widely used NZ quantification methods. Contrary to the hypothesis, the most important finding of this study was that NZ parameters lacked consistency and agreement when calculated using different methods, highlighting a need to clearly describe calculation method when presenting this parameter. TL‐EEZ methods and DS‐ST methods were most comparable, while all other NZ method comparisons lacked statistically significant agreement and typically differed by more than 30%. Surprisingly, several methods lacked consistency, indicating that NZ magnitudes obtained from different methods neither correlated nor displayed similar absolute values. Viscoelastic curve profiles had lower consistency and agreement, which was expected since the transition from NZ to elastic zone is less prominent in these profiles. The TL‐EEZ and DS‐ST methods had the most comparable NZ magnitude values for both triphasic and viscoelastic loading profiles.

Whenever a novel method to quantify the NZ has been introduced in the literature, the ZL method has been the common reference for comparison.^8^, ^11^, ^29^ A benefit of the ZL method is that it does not require arbitrary user input. NZ parameters obtained from this method are known to be sensitive to changes in motion segment instability due to degeneration and this method is therefore commonly used in larger animal models and human cadaveric studies.^7^, ^10^ However, the ZL method is not ideal for studies using smaller motion segment models due to the lower dynamic range over which their load‐deflection curve exist.^14^, ^15^, ^30^ Reduced control of off‐axis coupled motions are also known to influence these load‐deflection profiles.^30^ The ZL method is particularly susceptible to variable NZ magnitude measurements when load‐deflection profiles contain long laxity regions. A small shift in the load (x‐axis) in either limb generates large differences with the y‐axis intersection and subsequently increases the variability of the NZ magnitude measurement. This would explain the large NZ magnitude variability seen with the ZL method for the triphasic cohort and it also provides a potential explanation for why this method is rarely employed for smaller animal models and caudal motion segments known to have a longer NZ and more triphasic shaped profile. Our results corresponding to such triphasic profiles containing long NZ laxity regions show that the ZL method was not interchangeable with any other method. However, this method does show mild proportional correlations with ST and DS methods. The TL and ST methods were developed to characterize the NZ of triphasic load‐deflection curves. The ST method has been criticized for requiring user input when defining the ST.^8^ The DS method was developed to eliminate biased user input while relying solely on a mathematical assessment of the load‐deflection curve. However, it depends heavily on the goodness of fit of the 10‐parameter DS function and it is inconsistently adopted in the literature, presumably due to this complexity. Stolworthy et al described a similar, logistic, dual‐inflection point model (DIP‐Boltzmann equation) as an alternate to the DS model; however, we did not apply that method in this study because the literature appears to references the DS method more frequently.^31^ Our results indicate that the DS method is applicable for long, “triphasic” load‐deflection curves, generating NZ magnitudes comparable to the ST method.

Contrary to expectations, differences between NZ quantification methods were dependent on load‐deflection profile shape (ie, method A generated different NZ parameters than method B and this difference depended on the load‐deflection profile). It was notable that the ZL method generated larger NZ magnitudes compared to ST and DS when applied to triphasic profiles; contrarily with viscoelastic profiles, the ZL method generated the smallest values. The TL and EEZ always produced larger NZ magnitudes than the ST and DS methods, where the TL method generated the largest NZ magnitude for both profile cohorts. The dependency of some methods on load‐deflection profiles was unexpected and future studies will be important to better understand the most reliable methods across different loading profiles, species, spinal regions, and degenerative/injury states.

A “gold standard” method to measure NZ parameters irrespective of the biomechanical test set up does not exist. In this study, the lack of NZ magnitude agreement between methods across both profile cohorts indicated that methods measured different NZ regions. The lack of agreement was striking with viscoelastic load‐deflection profiles, where all method comparisons yielded significant absolute differences, indicating that NZ magnitude measurements of load‐deflection curves without clear triphasic regions may not be considered interchangeable. With these profiles, NZ magnitude consistency was only achieved between DS‐ST methods. This method comparison yielded ICC agreeability when load‐deflection curves demonstrated clear triphasic regions. However, when assessing their limits of agreement, the range of differences between these methods exceeded 30%, highlighting that interchangeability may not be appropriate in isolated cases. TL‐EEZ methods yielded interchangeable NZ magnitude measurements (ICC agreement = 0.81; *P* = .006) for triphasic curves. The current study found that DS‐ZL methods were not correlated, which corroborates the findings of Smit et al, where intact human lumbar spines with load‐deflection profiles similar to our viscoelastic cohort were used.^8^ Gay et al reported high correlations between EEZ‐ZL with cadaveric lumbar functional spinal units.^11^ The method of correlation was unclear, nor was this result consistent with our findings for either profile cohort. A standard reference NZ quantification method should be applicable to any load‐deflection curve profile and also reliable at measuring changes in NZ magnitude from intact to injured conditions. While the ZL method is consistently employed in biomechanical studies using large motion segments (ie, ovine, bovine, or human cadaveric specimens), it is not ideal for smaller motion segments. This work shows that most methods may not be considered interchangeable, implying that the NZ quantification methodology should be clearly described in order to contextualize the result. Future investigations are also warranted to determine if any NZ calculation method is best able to identify differences in NZ from the intact condition to a treatment condition involving degeneration or injury to the disc.

NZ stiffness was defined for the purposes of this study as the slope of the secant line connecting NZ boundaries, which permitted standardization and comparisons between methods. No significant differences were observed between methods, illustrating that significant deviations in NZ magnitude have a relatively small impact on the linear slope between the NZ boundaries. Agreement was common between methods for both profile cohorts, with DS‐ST and ZL‐EEZ agreeing independent of the profile cohort. Despite this agreement, Bland‐Altman plots illustrate wide limits of agreement indicating that interchanging NZ stiffness measures between methods should be approached with caution. In practice, comparing NZ stiffness across studies is rare, since stiffness measures are often defined within a study and compared internally across treatment groups.

Investigation into the validity of treating NZ stiffness as a linear slope between NZ boundaries yielded no superior NZ quantification method. We note that the triphasic profile cohort exhibited a more nonlinear NZ region than the viscoelastic profile, since the linear model R^2^ values were less than 0.7 and 0.9 for triphasic and viscoelastic profiles, respectively. The DS method constrained NZ boundaries with the most linear load‐deflection relationship when applied to triphasic profile, although this finding was not significantly different from the other methods. In the literature, NZ stiffness has been reported as a tangent slope at the point of zero load, or point of zero deflection, or at the curve's inflection point. We show that for triphasic profiles, a single value for NZ stiffness is not as accurate as a nonlinear description. However, this study also showed that measuring NZ stiffness as single secant across NZ boundaries does yield comparable measures across profile types that were independent of the method and thus can be used as a reasonable approximation.

There were some limitations of this study. Only load‐deflection curves of rat motion segments were studied and therefore neither profile fully represents the diverse spectrum of load‐deflection profiles from different species or states of injury and degeneration. However, since we compared two datasets with highly distinct profiles, our conclusion that comparability of NZ quantification methods varies with load‐deflection profiles is expected to remain valid across a large range of conditions. Therefore, irrespective of the input conditions used to test a specimen of species “X” which generate load‐defection curves with profile “Y,” this study shows that the calculated NZ value of profile “Y” will depend on what method is chosen to make that calculation. We tested motion segments under a stiffness protocol because the low forces and small deformations in rat motion segments required improved biomechanical test system control. We, therefore, transformed our stiffness data to the flexibility configuration, when applying our data to NZ quantification methods requiring flexibility data (ie, compliance configuration). This study highlights a need for increased transparency to ensure that NZ quantification methods are described clearly in the literature. With a clear lack of consistency across methods, we also believe it is premature to claim there is a single optimal method especially since there is no uniformly applied standard reference method in the literature. Future studies are required to assess how NZ quantification methods differ when applied to load‐deflection profiles from other animal and human models and across treatment conditions.

This study shows that differences exist between NZ quantification methods and that agreement between methods is less than expected. Comparisons between DS‐ST and TL‐EEZ methods exhibited the highest degree of interchangeability for both load‐deflection profiles. This study also determined that consistency and agreement between methods was dependent on the load‐deflection profile with viscoelastic curve profiles showing greater disagreement. The lack of agreement among methods highlights the need to describe the methodology when reporting NZ results. This study also highlights a need for future studies to determine if a reference standard method exists that can best identify NZ changes across degeneration, injury, or repair conditions in different species.

## AUTHOR CONTRIBUTIONS

Theodor Di Pauli von Treuheim initiated the project, generated the Matlab program, developed methods, analyzed data, and wrote first draft of the manuscript. Olivia M. Torre initiated the project, performed biomechanical testing of rat caudal specimen, and contributed to data analysis, interpretation of results, and manuscript writing. Grace E. Mosley performed biomechanical testing of rat lumbar specimen and contributed to interpretation of results, and manuscript writing. Phil Nasser contributed to biomechanical testing, data analysis, interpretation of results, and manuscript writing. James C. Iatridis contributed to project initiation, methods development, interpretation of results, and manuscript writing.

## Supporting information


**Figure S1**. Supporting details for Figure 2.
