# Supplementary figures and images for: Measuring the neutral zone of spinal motion segments: Comparison of multiple analysis methods to quantify spinal instability
Source: JOR Spine. 2020 Apr 25;3(2):e1088. doi: 10.1002/jsp2.1088 (PMC7323462; doi:10.1002/jsp2.1088)

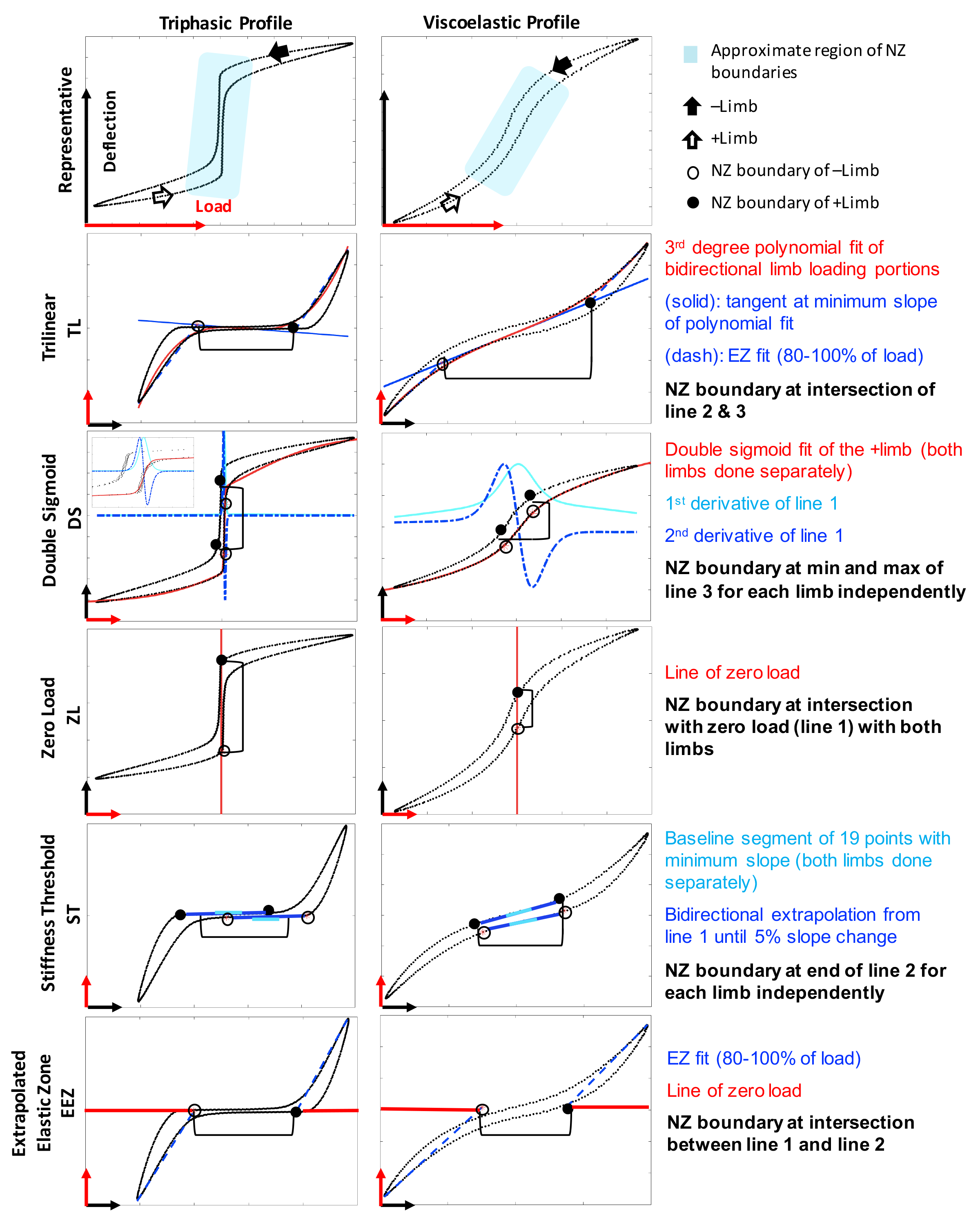

Supplement: Supplementary file 1 — Figure S1. Supporting details for Figure 2. [file JSP2-3-e1088-s001.tif]
